# Evaluation of Biomechanical Stability of Teeth Tissue According to Crown Materials: A Three-Dimensional Finite Element Analysis

**DOI:** 10.3390/ma16134756

**Published:** 2023-06-30

**Authors:** Youngjae Yoon, Myung-Jin Lee, Inyeong Kang, Sanghwan Oh

**Affiliations:** 1 Department of Oral and Maxillofacial Surgery, School of Dentistry, Kyung Hee University, Seoul 02447, Republic of Korea; 2 Department of Dental Hygiene, Division of Health Science, Baekseok University, Cheonan 31065, Republic of Korea; 3 School of Mechanical Engineering, Korea University, Seoul 02841, Republic of Korea; 4 Department of Dental Hygiene, Konyang University, Daejeon 35365, Republic of Korea

**Keywords:** crown materials, finite element analysis, stability

## Abstract

The biomechanical effects of dental tissue according to various dental crown materials were investigated using finite element analysis. Bone, prepared tooth, root canal, and periodontal ligament were modeled based on computed tomography. Depending on the characteristics of the crown material, it was classified into zirconia, hybrid ceramic, gold alloy, and acrylic resin. A loading force of 200 N was applied in the vertical direction to the occlusal surface of the crown, and analysis was performed under the condition that all interfaces were tied. The results demonstrate that the highest von Mises stress was shown in the prepared tooth of the acrylic resin model, which is a temporary prosthesis, and the pulpal pressure was also the highest. Additionally, among the final prosthesis, the highest stress was shown in the hybrid ceramic model prepared teeth. The properties of restoration materials can be a factor influencing the tooth structure. Thus, in order to make a correct decision when selecting a material for restorative treatment, it is necessary to understand, analyze, and evaluate the properties of these restoration materials.

## 1. Introduction

Preservation of healthy teeth is an important factor in maintaining physical, mental, and social health. In recent years, functional and aesthetic aspects have gained importance in dental restorative treatment, and the demand for advanced dental treatment that can satisfy both simultaneously is increasing [1,2,3]. Dental crowns have been widely used in prosthetic treatment due to their high level of accuracy and excellent durability [4,5]. In dental restorative materials, gold has stable biocompatibility [6,7,8]. Ceramics have excellent aesthetics, and zirconia shows the highest flexural strength and fracture toughness among the existing ceramic materials [9,10]. Despite their many advantages, dental crowns are potentially susceptible to mechanical failure, which can lead to catastrophic fractures and impact tooth structure [11]. Successful tooth restoration has a great impact on tooth survival. The type of dental restorative material is an important factor related to the success of restoration. Several previous studies have investigated materials used for restorative treatment and reported the use of zirconia, ceramics, alloys, nanocomposites, and hybrid materials. Ha et al. [12] evaluated the stress distribution in the posterior monolithic zirconia crowns with different cement thicknesses under masticatory force and maximum bite force using three-dimensional finite element analysis. Madruga et al. [13] proposed a new approach for crown veneers using an aesthetic porcelain coating only in part of the zirconia infrastructure and reported that the highly translucent zirconia ceramic only associated with the buccal covering. Ceramic could add aesthetic gain and rigidity to the system and could be a good option while restoring maxillary molars in patients who do not have parafunction. Sichi et al. [14] evaluated the effect of a biologically oriented preparation technique on the stress concentration of endodontically treated upper central incisors restored with zirconia crown. Kim et al. [15] analyzed the stress distribution and crack propagation in identical cracked tooth models after treatment with various materials and designs. In most previous studies, the reason for using each material was not investigated in depth, and only the stress on the crown or cement layer on the endodontically treated tooth was evaluated. There is a lack of studies evaluating the stress on dental structures such as pulp and periodontal ligament in vital tooth restoration treatment. In order to function properly in the oral environment, restorative materials must meet the biological, esthetic, and mechanical requirements such as resistance to masticatory loads [16]. Numerical simulations can be performed using finite element analysis (FEA) to understand the biomechanical properties of the restorative material and the effect of the restorative material on the tooth tissues. This method has been extensively reported in the literature for evaluating restorative materials [17,18,19]. FEA can identify problems by evaluating the stress and deformation states of the material and by evaluating the stress distribution generated by the chewing load through model standardization to provide an acceptable approximate solution [20]. Therefore, the purpose of this study is to analyze and evaluate the biomechanical effects in tooth tissue with various dental crown materials using FEA.

## 2. Materials and Methods

**Figure 1:**
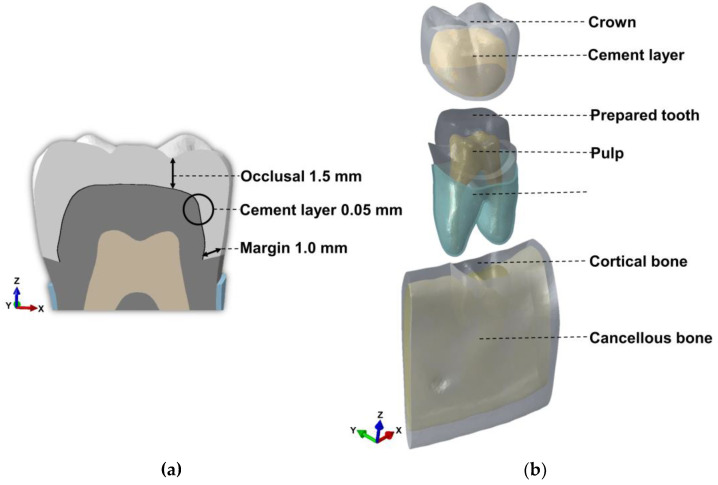
(**a**) Thickness of crown and cement layer; (**b**) components of the final model.

Based on computed tomography (CT) in transverse plane with a total of 392 slices of 0.75 mm thick slices, a three-dimensional (3D) model of the human mandibular bone was generated using Mimics software v17.0. First, the entire mandibular bone and cancellous bone except the teeth were segmented, and then the enamel, dentin, and pulp of the 1st molar were segmented. The segmented 3D model was smoothed through the smoothing module of mimics software v9.0 and the local smoothing module of 3-matic software v9.0; the mandibular bone was composed of cortical and cancellous bone, and the 1st molar was enamel, dentin, pulp, and periodontal ligament (PDL). For the segmentation of cortical and cancellous bone, cortical bone was obtained by subtracting cancellous bone from the entire mandible using the Boolean subtraction module of 3-matic software v9.0. Next, based on the 3D enamel model of the tooth, a crown with a margin of about 1 mm and an occlusal thickness of about 1.5 mm was made, and a prepared tooth model was made based on dentine (Figure 1a) [21]. The internal layer of the crown was subsequently duplicated and used as a base for making the cementing line with 0.05 mm of thickness (Figure 1a) [22]. The components of the final model include cortical bone, cancellous bone, crown, cement layer, prepared tooth, pulp, and PDL (Figure 1b).

**Figure 2:**
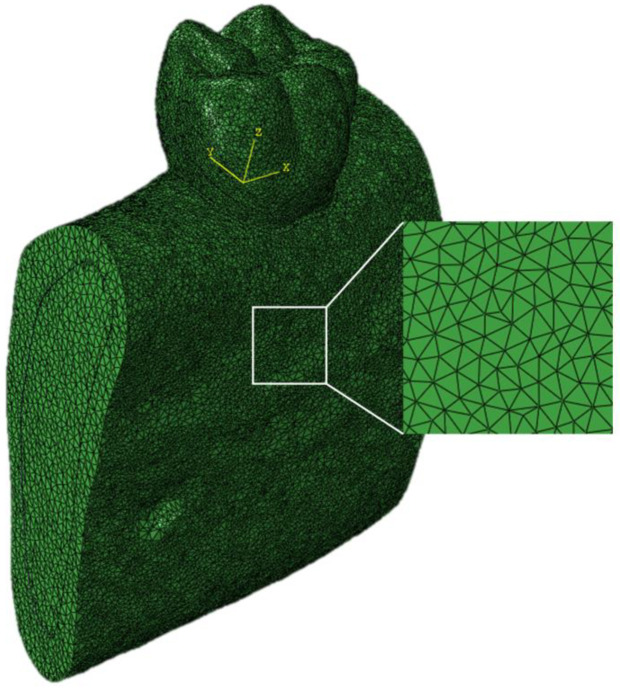
Mesh construction of the model.

By using a 3-matic software, v9.0 remesh module, a surface and volume mesh for FEA was constructed. The mandible consisted of 1.0 mm element size, and the tooth was 0.5 mm element size. The mesh consisted of four-node tetrahedral elements (Figure 2). The total number of nodes and elements for components of the model is listed in Table 1. All surfaces and volume mesh were imported to the FEA software ABAQUS v6.14.

The material properties were assumed to be homogeneous, isotropic, and linearly elastic. The crown types used in the study were classified as zirconia, ceramic, gold alloy, and acrylic resin based on the suggestions by previous research. The material properties of resin cement were used for the cement layer of zirconia, ceramic, and gold alloy crowns, and temporary cement were used for the cement layer of acrylic resin crown. The material properties of the cortical bone, cancellous bone, prepared tooth, pulp, PDL, crown, and cement layer used in the analysis are shown in Table 2.

**Figure 3:**
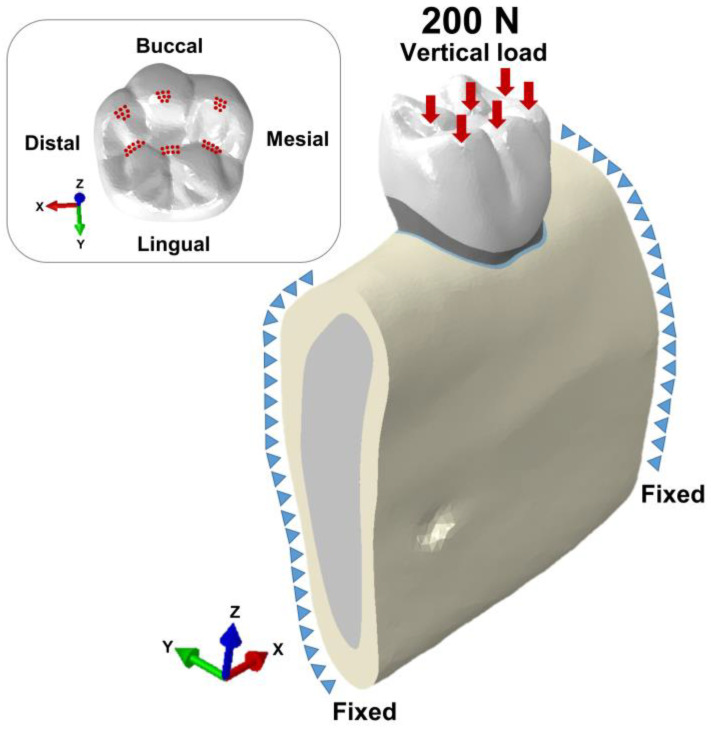
Boundary and loading conditions.

As shown in Figure 3, a vertical load of maximum biting force 200 N was applied to a total of 50 nodes, and a force of 4 N per node was applied [26]. Tie conditions were applied between the interface of all parts, and contact conditions were not considered. Both sides of the cortical and cancellous bone sections were fixed in all directions (X, Y, Z).

## 3. Results

### 3.1. Total Strain Energy

**Figure 4:**
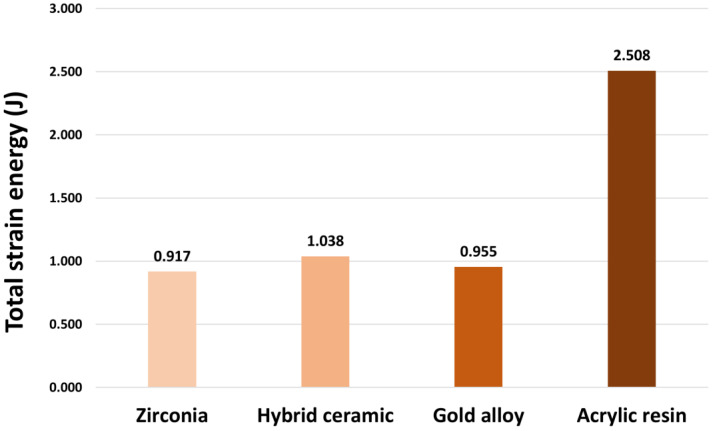
The total strain energy values of each model.

The total strain energy of the structure under the prescribed loading and boundary conditions is an effective global stiffness measure with lower strain energy indicating higher stiffness. The whole model stiffness during the simulation was estimated by measuring the total strain energy. Figure 4 shows the analysis results of the strain energy values on the whole model. The model with the highest strain energy measured was the acrylic resin model (2.51 J) combined with the tooth with temporary cement, and the highest energy was measured in the following order: hybrid ceramic (1.04 J), gold alloy (0.95 J), and zirconia (0.92 J).

### 3.2. Pressure on the Pulp

**Figure 5:**
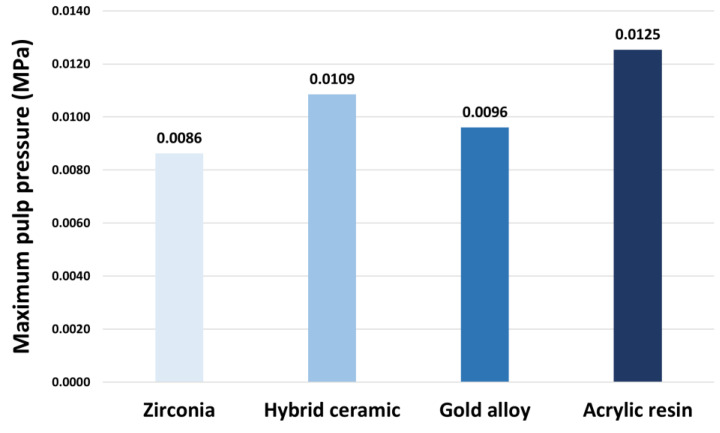
The maximum pressure values of pulp.

**Figure 6:**
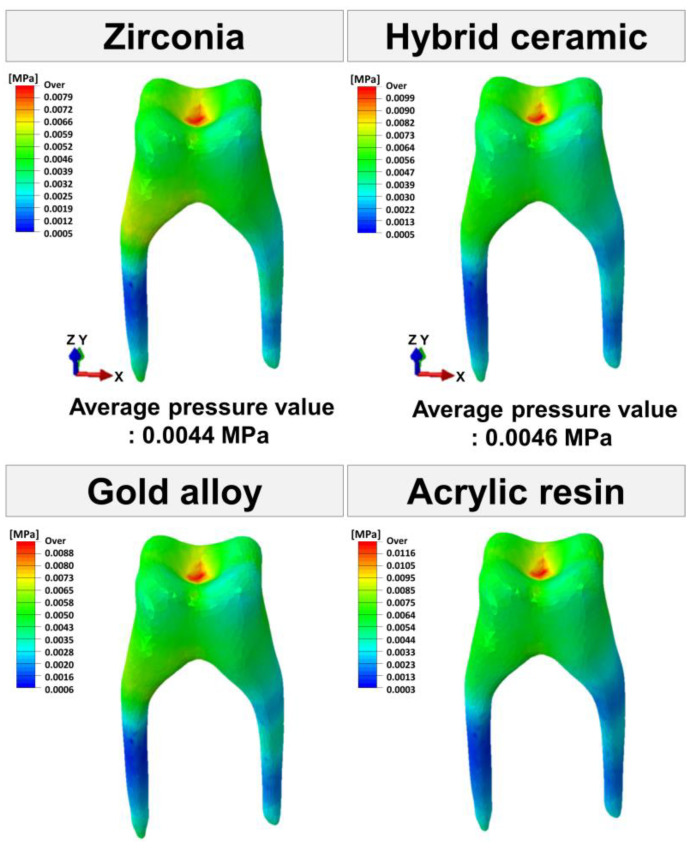
The pressure (MPa) distribution in pulp.

The maximum and average pressure values applied to the pulp were calculated (Figure 5 and Figure 6). The average pressure value did not show a significant difference, and the maximum pressure value was higher in the order: acrylic resin (0.0125 MPa), hybrid ceramic (0.0109 MPa), gold alloy (0.0096 MPa), and zirconia (0.0086 MPa).

### 3.3. Von Mises Stress

**Figure 7:**
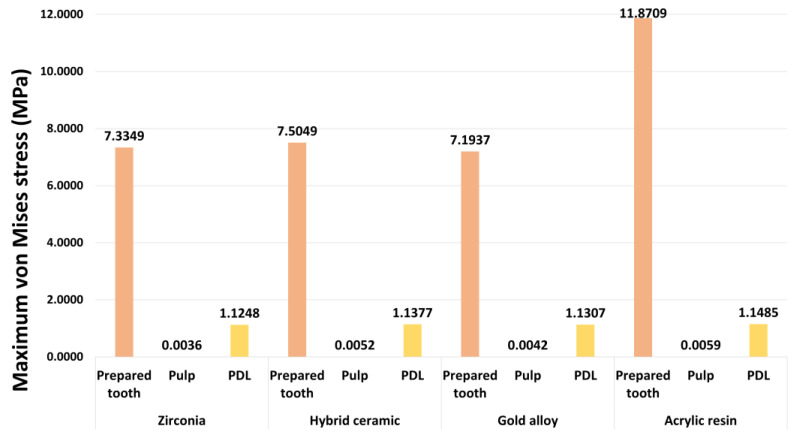
The maximum von Mises stress values of each model.

**Figure 8:**
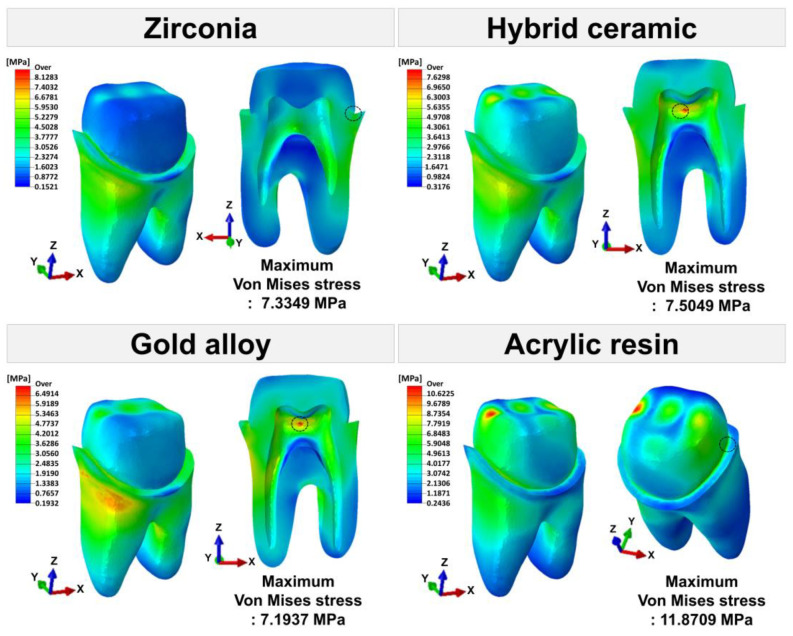
The von Mises stress (MPa) distribution in prepared tooth. The area indicated by the black dotted line is the area where the maximum von Mises stress appears.

**Figure 9:**
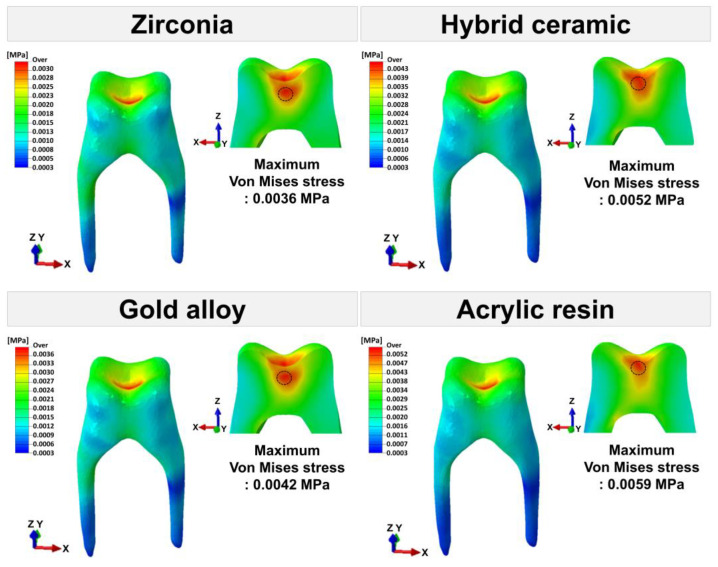
The von Mises stress (MPa) distribution in pulp. The area indicated by the black dotted line is the area where the maximum von Mises stress appears.

**Figure 10:**
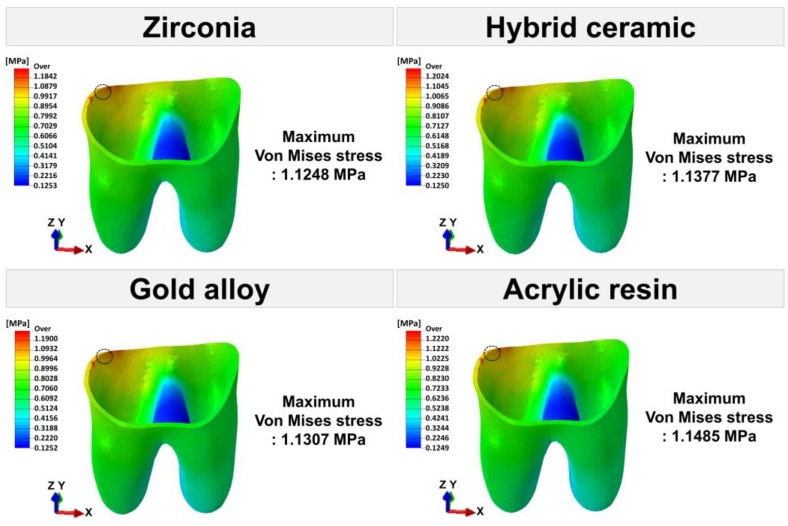
The von Mises stress (MPa) distribution in PDL. The area indicated by the black dotted line is the area where the maximum von Mises stress appears.

The maximum von Mises stress values for each tooth tissue are presented in Figure 7. Figure 8, Figure 9 and Figure 10 show the chromatic presentation of the von Mises stress distribution in each tooth tissue and location of the maximum value according to crown material.

The model with the highest stress in the tooth tissue was the acrylic resin model. Among them, the highest stress value was shown in the prepared tooth (11.8709 MPa). The prepared tooth of the acrylic resin model showed about 1.6 times higher stress values than those of the other models. In the other models, high stress values occurred in the following order: hybrid ceramic (7.5049 MPa), zirconia (7.3349 MPa), and gold alloy (7.1937 MPa). The position of the maximum stress value of the prepared teeth was different for each model. The maximum stress value occurred at the mesial margin in the zirconia model, the pulp chamber region in the hybrid ceramic and gold alloy model, and the distal margin in the acrylic resin model (Figure 5). In pulp, the highest stress value occurred in the acrylic resin model (0.0059 MPa), followed by hybrid ceramic (0.0052 MPa), gold alloy (0.0042 MPa), and zirconia (0.0036 MPa). The area where the maximum stress value appeared in the pulp was the pulp chamber, which was similar in all models (Figure 5). In PDL, the highest stress value occurred in the acrylic resin model (1.1485 MPa), and, like pulp, the stress value was high in the following order: hybrid ceramic (1.1377 MPa), gold alloy (1.1307 MPa), and zirconia (1.1248 MPa). The area where the maximum stress value appeared in PDL was the top of the mesial and was similar in all models (Figure 6). In addition, in all crown models, the highest stress values appeared in the following order: prepared tooth, PDL, and pulp.

## 4. Discussion

Rehabilitation of teeth with extensive damage is one of the challenging tasks in restorative dentistry [27]. The most common method is to restore the entire crown. Crown restorations have been universally and widely used. Materials used for restorative treatment have different characteristics from natural teeth. Because the mechanical properties between the material and the tooth tissue are different, problems may occur when exposed to load conditions such as masticatory force related to the patient’s habit and finally weaken the tooth tissue to affect the survival of the tooth [28,29,30]. Several studies have investigated the materials used for restorative treatment [12,13,14,15]. However, in most of the studies, the stress distribution in the endo-crown was analyzed, and the analysis of the tooth tissue in the vital teeth was not performed [17,27,31,32].

In this study, FEA was used to analyze and evaluate the biomechanical effects of various dental crown materials on tooth tissue. Numerous studies have adopted FEA as a method for designing, modifying, or validating new systems and materials prior to conducting in vivo testing [20]. Additionally, FEA has been widely applied in dental biomechanical studies to investigate the stresses generated in tooth tissue and to predict the clinical performance of restorations [33,34]. The material of the crown used in the study consisted of acrylic resin for the temporary prosthesis, and zirconia, hybrid ceramic, and gold alloy for the final prosthesis. In addition, to reproduce the oral anatomical structure as much as possible, the model was divided into prepared teeth, pulp, PDL, cortical bone, and cancellous bone. Surface and volume meshes were constructed for each structure, and the material properties were assumed to be homogeneous, isotropic, and linearly elastic. As per the results of this study, the total strain energy was the highest in the dental model restored with acrylic resin. The total strain energy of the structure under the prescribed loading and boundary conditions is an effective global stiffness measure with lower strain energy indicating higher stiffness. This means that the acrylic resin model is the most fragile. Von Mises stress was analyzed according to the tooth tissue of each model, and the highest stress occurred in the prepared tooth of the acrylic resin model. The pressure applied to the pulp also showed the highest pressure value in the acrylic resin model. The use of temporary restorations is an essential and important part of the dental restorative treatment procedure. Because provisional restorations are very useful for maintaining proper oral function during the final prosthesis manufacturing period and until the final restoration is fitted, it can directly affect the success of the final restoration [35]. However, according to the results of this study, acrylic resin crowns, which are temporary restorations, concentrate stress on the tooth tissue together with temporary cement. Previous studies reported that temporary crowns and adhesives have high resistance as temporary materials; however, when stress is concentrated, they cause cohesive failure of the cement layer, and these defects can damage the tooth structure in the long term [36,37,38,39]. Among zirconia, hybrid ceramic, and gold alloy models of the final prosthesis, the highest stress was shown in the tooth tissue of the hybrid ceramic. The maximum pressure of the pulp was the highest in hybrid ceramic, followed by gold and zirconia. Previous studies have reported that ceramic crowns are biologically superior to other metallic restorations; however, due to the mechanical properties of the material, they cause cracks in the ceramic structure when a load is applied, leading to failure in terms of chipping or complete destruction [40]. Finally, the material properties of the restoration can be a factor influencing the tooth structure, which means that there is an influence between the elastic modulus of the material and the stress distribution on the tooth structure. A material with a high modulus of elasticity transmits less stress to protect healthy tooth tissue, and a material with a low modulus of elasticity transmits more stress to tooth tissue.

A limitation of this study is that it could not fully simulate in vivo conditions. Dental structures including bones were modeled; however, soft tissues such as the gingiva and muscles acting during chewing were not considered. In addition, this study analyzed the stress distribution on the crown of the first molar only under static load. In the future, studies on various loads or fatigue loads are needed. Although in vitro studies such as FEA are limited because they do not completely mimic the intraoral environment, these studies provide a baseline for further clinical evaluation. Biomechanical simulation of restorations can be a useful tool for researchers trying to develop restoration designs. In addition, by utilizing the analyzed stress distribution pattern, it is possible to predict the range and location of stress concentration in various restorative materials and designs used in clinical practice.

## 5. Conclusions

Biting force is transmitted to the tooth tissue and causes compression. It is important to study the measurements delivered to the tooth tissue at a maximum biting force reflecting oral conditions. According to the results of this study, the properties of the restoration material can be a factor affecting the tooth tissue. The lower the Young’s modulus of the material, the higher the stress value. The stress applied to the tooth tissue is transmitted to the bone and may cause bone resorption in the long term. Thus, to make a correct decision when selecting materials for restorative treatment, it is necessary to understand the characteristics of these restorative materials to predict and analyze the stress distribution, along with other properties such as aesthetics and fatigue resistance. Finally, understanding these characteristics can increase the success rate of long-term treatment.

## Figures and Tables

**Table 1:** Number of nodes and elements for components of the model.

Composition of the Model	Nodes	Element
Cortical bone	55,262	232,995
Cancellous bone	41,847	206,701
Crown	20,442	106,634
Cement layer	4508	13,883
Prepared tooth	40,607	221,363
Pulp	8480	41,127
Periodontal ligament	7540	23,698

**Table 2:** Material properties for components of model.

Composition of the Model		Young’s Modulus (MPa)	Poisson’s Ratio
Cortical bone [23]		13,700	0.3
Cancellous bone [23]		1370	0.3
Prepared tooth [24]		18,600	0.31
Pulp [24]		6.8	0.45
Periodontal ligament [24]		70	0.45
Crown	Zirconia [8]	200,000	0.31
	Hybrid ceramic [8]	34,700	0.28
	Gold alloy [8]	91,000	0.32
	Acrylic resin [25]	2200	0.3
Cement layer	Resin cement [8]	7500	0.3
	Temporary cement [25]	1350	0.3

## Data Availability

Not applicable.

## References

[1] Attard N.J., Zarb G.A. (2004). Long-term treatment outcomes in edentulous patients with implant-fixed prostheses: The Toronto study. Int. J. Prosthodont..

[2] Sevimay M., Turhan F., Kilicarslan M.A., Eskitascioglu G. (2005). Three-dimensional finite element analysis of the effect of different bone quality on stress distribution in an implant-supported crown. J. Prosthet. Dent..

[3] Nicolaisen M.H., Bahrami G., Finlay S., Isidor F. (2014). Comparison of fatigue resistance and failure modes between metal-ceramic and all-ceramic crowns by cyclic loading in water. J. Dent..

[4] Behr M., Winklhofer C., Schreier M., Zeman F., Kobeck C., Brauer I., Rosentritt M. (2012). Risk of chipping or facings failure of metal ceramic fixed partial prostheses—A retrospective data record analysis. Clin. Oral Investig..

[5] Katzenbach A., Dorsam I., Stark H., Bourauel C., Keilig L. (2021). Fatigue behaviour of dental crowns made from a novel high-performance polymer PEKK. Clin. Oral Investig..

[6] Pjetursson B.E., Sailer I., Makarov N.A., Zwahlen M., Thoma D.S. (2015). All-ceramic or metal-ceramic tooth-supported fixed dental prostheses (FDPs)? A systematic review of the survival and complication rates. Part II: Multiple-unit FDPs. Dent. Mater..

[7] Sailer I., Makarov N.A., Thoma D.S., Zwahlen M., Pjetursson B.E. (2015). All-ceramic or metal-ceramic tooth-supported fixed dental prostheses (FDPs)? A systematic review of the survival and complication rates. Part I: Single crowns (SCs). Dent. Mater..

[8] Dal Piva A.M.O., Tribst J.P.M., Borges A.L.S., Souza R., Bottino M.A. (2018). CAD-FEA modeling and analysis of different full crown monolithic restorations. Dent. Mater..

[9] Pittayachawan P., McDonald A., Young A., Knowles J.C. (2009). Flexural strength, fatigue life, and stress-induced phase transformation study of Y-TZP dental ceramic. J. Biomed. Mater. Res. Part B Appl. Biomater..

[10] Liu B., Lu C., Wu Y., Zhang X., Arola D., Zhang D. (2011). The effects of adhesive type and thickness on stress distribution in molars restored with all-ceramic crowns. J. Prosthodont..

[11] Ibrahim R.O., Al-Zahawi A.R., Sabri L.A. (2021). Mechanical and thermal stress evaluation of PEEK prefabricated post with different head design in endodontically treated tooth: 3D-finite element analysis. Dent. Mater. J..

[12] Seung-Ryong Ha S.-H.K., Lee J.-B., Han J.-S., Yeo I.-S., Yoo S.-H., Kim H.-K. (2016). Biomechanical three-dimensional finite element analysis of monolithic zirconia crown with different cement thickness. Ceram. Int..

[13] Camila Ferreira Leite Madruga G.F.R., Borges A.L.S., de Siqueira Ferreira Anzaloni Saavedra G., Souza R.O., de Melo Marinho R.M., Penteado M.M. (2021). Stress Distribution in Modified Veneer Crowns: 3D Finite Element Analysis. Oral.

[14] Sichi L.G.B., Pierre F.Z., Arcila L.V.C., de Andrade G.S., Tribst J.P.M., Ausiello P., di Lauro A.E., Borges A.L.S. (2021). Effect of Biologically Oriented Preparation Technique on the Stress Concentration of Endodontically Treated Upper Central Incisor Restored with Zirconia Crown: 3D-FEA. Molecules.

[15] Kim S.Y., Kim B.S., Kim H., Cho S.Y. (2021). Occlusal stress distribution and remaining crack propagation of a cracked tooth treated with different materials and designs: 3D finite element analysis. Dent. Mater..

[16] Keys W.F., Keirby N., Ricketts D.N.J. (2016). Provisional Restorations—A Permanent Problem?. Dent. Update.

[17] da Fonseca G.F., de Andrade G.S., Dal Piva A.M.O., Tribst J.P.M., Borges A.L.S. (2018). Computer-aided design finite element modeling of different approaches to rehabilitate endodontically treated teeth. J. Indian Prosthodont. Soc..

[18] Helal M.A., Wang Z. (2019). Biomechanical Assessment of Restored Mandibular Molar by Endocrown in Comparison to a Glass Fiber Post-Retained Conventional Crown: 3D Finite Element Analysis. J. Prosthodont..

[19] Ghoul W.E., Ozcan M., Tribst J.P.M., Salameh Z. (2020). Fracture resistance, failure mode and stress concentration in a modified endocrown design. Biomater. Investig. Dent..

[20] Ausiello P., Ciaramella S., De Benedictis A., Lanzotti A., Tribst J.P.M., Watts D.C. (2021). The use of different adhesive filling material and mass combinations to restore class II cavities under loading and shrinkage effects: A 3D-FEA. Comput. Methods Biomech. Biomed. Eng..

[21] Shahmoradi M., Wan B., Zhang Z., Wilson T., Swain M., Li Q. (2020). Monolithic crowns fracture analysis: The effect of material properties, cusp angle and crown thickness. Dent. Mater..

[22] Ha S.R. (2015). Biomechanical three-dimensional finite element analysis of monolithic zirconia crown with different cement type. J. Adv. Prosthodont..

[23] Madfa A.A., Al-Hamzi M.A., Al-Sanabani F.A., Al-Qudaimi N.H., Yue X.G. (2015). 3D FEA of cemented glass fiber and cast posts with various dental cements in a maxillary central incisor. Springerplus.

[24] Zhu J., Rong Q., Wang X., Gao X. (2017). Influence of remaining tooth structure and restorative material type on stress distribution in endodontically treated maxillary premolars: A finite element analysis. J. Prosthet. Dent..

[25] Tribst J.P.M., Borges A.L.S., Silva-Concilio L.R., Bottino M.A., Ozcan M. (2021). Effect of Restorative Material on Mechanical Response of Provisional Endocrowns: A 3D-FEA Study. Materials.

[26] Hu J., Dai N., Bao Y., Gu W., Ma J., Zhang F. (2013). Effect of different coping designs on all-ceramic crown stress distribution: A finite element analysis. Dent. Mater..

[27] Zhang Y., Lai H., Meng Q., Gong Q., Tong Z. (2022). The synergetic effect of pulp chamber extension depth and occlusal thickness on stress distribution of molar endocrowns: A 3-dimensional finite element analysis. J. Mater. Sci. Mater. Med..

[28] Govare N., Contrepois M. (2020). Endocrowns: A systematic review. J. Prosthet. Dent..

[29] Azeem U.L., Yaqin Syed D.R., Shirin S., Nicolas M. (2021). Three-dimensional finite element analysis of stress distribution in a tooth restored with full coverage machined polymer crown. Appl. Sci..

[30] Moga R.A., Buru S.M., Olteanu C.D. (2022). Assessment of the Best FEA Failure Criteria (Part I): Investigation of the Biomechanical Behavior of PDL in Intact and Reduced Periodontium. Int. J. Environ. Res. Public Health.

[31] Shams A., Sakrana A.A., Abo El-Farag S.A., Elerian F.A., Ozcan M. (2021). Biomechanical behavior of endodontically treated premolar teeth restored with novel endocrown system: 3D Finite Element and Weibull analyses. J. Mech. Behav. Biomed. Mater..

[32] Zheng Z., He Y., Ruan W., Ling Z., Zheng C., Gai Y., Yan W. (2021). Biomechanical behavior of endocrown restorations with different CAD-CAM materials: A 3D finite element and in vitro analysis. J. Prosthet. Dent..

[33] Tribst J.P.M., Dal Piva A.M.O., Madruga C.F.L., Valera M.C., Borges A.L.S., Bresciani E., de Melo R.M. (2018). Endocrown restorations: Influence of dental remnant and restorative material on stress distribution. Dent. Mater..

[34] Tribst J.P.M., Lo Giudice R., Dos Santos A.F.C., Borges A.L.S., Silva-Concilio L.R., Amaral M., Lo Giudice G. (2021). Lithium Disilicate Ceramic Endocrown Biomechanical Response According to Different Pulp Chamber Extension Angles and Filling Materials. Materials.

[35] Balkenhol M., Mautner M.C., Ferger P., Wostmann B. (2008). Mechanical properties of provisional crown and bridge materials: Chemical-curing versus dual-curing systems. J. Dent..

[36] Hill E.E., Lott J. (2011). A clinically focused discussion of luting materials. Aust. Dent. J..

[37] Carmello J.C., Fais L.M., Ribeiro L.N., Claro Neto S., Guaglianoni D.G., Pinelli L.A. (2012). Diametral tensile strength and film thickness of an experimental dental luting agent derived from castor oil. J. Appl. Oral Sci..

[38] Campaner L.M., Silveira M.P.M., de Andrade G.S., Borges A.L.S., Bottino M.A., Dal Piva A.M.O., Lo Giudice R., Ausiello P., Tribst J.P.M. (2021). Influence of Polymeric Restorative Materials on the Stress Distribution in Posterior Fixed Partial Dentures: 3D Finite Element Analysis. Polymers.

[39] Benazzi S., Nguyen H.N., Kullmer O., Kupczik K. (2016). Dynamic Modelling of Tooth Deformation Using Occlusal Kinematics and Finite Element Analysis. PLoS ONE.

[40] Zhang Y., Sailer I., Lawn B.R. (2013). Fatigue of dental ceramics. J. Dent..

